# Cardiovascular risk factors in adult general out-patient clinics in Nigeria: a country analysis of the Africa and Middle East Cardiovascular Epidemiological (ACE) study

**DOI:** 10.4314/ahs.v17i4.15

**Published:** 2017-12

**Authors:** Geoffrey C Onyemelukwe, Oluwagbenga Ogunfowokan, Amam Mbakwem, A Kayode Alao, Kodjo Soroh, Osahon Omorodion, Paula Abreu

**Affiliations:** 1 Department of Medicine, Ahmadu Bello University Teaching Hospital Zaria, Zaria, Nigeria; 2 Department of Family Medicine, National Hospital Abuja, Abuja, Nigeria; 3 Department of Medicine, Lagos University Teaching Hospital, Lagos, Nigeria; 4 Department of Family Medicine, Federal Medical Centre Keffi, Nigeria; 5 Pfizer Specialties Ltd, Nigeria/East Africa Region, Lagos, Nigeria; 6 Pfizer Inc, New York, NY, USA

**Keywords:** Nigeria, cardiovascular risk factors, screening programs, risk factor management, The Africa and Middle East Cardiovascular Epidemiological (ACE) study

## Abstract

**Background:**

With globalization and rapid urbanization, demographic and epidemiologic transitions have become important determinants for the emergence of cardiovascular disease (CVD).

**Objective:**

To estimate the prevalence of CVD risk factors in adult out-patients attending general practice and non-specialist clinics in urban and rural Nigeria.

**Methods:**

As part of the Africa and Middle East Cardiovascular Epidemiological (ACE) study, a cross-sectional epidemiologic study was undertaken for the presence of hypertension, diabetes mellitus, dyslipidemia, obesity, smoking and abdominal obesity in Nigeria.

**Results:**

In total, 303 subjects from 8 out-patient general practice clinics were studied, 184 (60.7%) were female and 119 (39.3%) were male. Mean age was 42.7±13.1 years; 51.8% were aged <45 years; 4% ≥65 years. Over 90% of subjects had ≥1 of 6 selected modifiable cardiovascular risk factors: 138 (45.6%) had 1–2; 65 (21.5%) had 3; 60 (19.8%) had 4; and 11 (3.6%) had 5 concurrent risk factors. Screening identified 206 subjects (68.0%) with dyslipidemia who did not have a prior diagnosis.

**Conclusion:**

Cardiovascular risk factors are highly prevalent in Nigerian subjects attending out-patient clinics. Moreover, many subjects were undiagnosed and therefore unaware of their cardiovascular risk status. Opportunistic screening alongside intensive national, multisectoral education or risk factor education is needed, should be scaled up nationwide and rolled out in both urban and rural communities in Nigeria.

## Background

Cardiovascular disease (CVD) has emerged in recent decades as a major cause of morbidity and mortality worldwide^1^–^3^. An estimated 17-million deaths globally were due to CVD in 2002, and CVD or stroke are projected to become the worldwide leading cause of morbidity and mortality by 2020^3^. This projection applies especially to low and middle-income countries, including Nigeria. Countries like Nigeria are undergoing major demographic transition associated with a progressive ageing population, as well as epidemiologic transition.

Throughout the 1950s and 1960s, hypertension was relatively rare in African nations^4^, but in recent decades the prevalence has increased dramatically. For example, in a 1979 Nigerian National Survey the prevalence of hypertension was approximately 11%, using a cut-off for diagnosis of blood pressure (BP) ≥160/95 mm Hg^5^. Using the conventional cut-off (BP ≥140/90 mm Hg) prevalence was nearly 20% in a national survey carried out in 2003 using a WHO step-wise questionnaire^6^. The rarity of large vessel disease in West Africans has been attributed not only to a non-Western diet, but also to the beneficial characteristics of platelets which, unlike those of Europeans, easily undergo spontaneous disaggregation when aggregators are present, in addition to spontaneous rapid fibrinolysis^7^. According to Burkitt^8^, these protective advantages of platelets are gradually being eroded by westernized lifestyles, and other practices, such as smoking, lack of exercise, urbanization and an excessive consumption of sugary drinks^9^.

Furthermore, the advantages afforded by spontaneous disaggregation in Nigerians who have not adopted Western lifestyles appear to be lost in Nigerians with diabetes mellitus^10^. Consequently, the emergence of diabetes and other cardiovascular risk factors^11^, including the metabolic syndrome^12^, together with coronary artery disease^13^–^15^, stroke^16^,^17^, cardiovascular morbidities and mortalities have become serious healthcare burdens in Nigeria^18^–^20^. As a result, systematic studies have been initiated alongside adoption of random risk factor screening program in healthcare clinics, in order to encourage healthier lifestyles in both rural and urban Nigerian communities. The Africa and Middle East Cardiovascular Epidemiological (ACE) Study^21^ was undertaken with the objective of estimating the prevalence of cardiovascular risk factors in out-patients attending general practice and non-specialist clinics in urban and rural communities, including in Nigeria.

## Methods

The ACE study (July 2011 to April 2012) was a cross-sectional epidemiologic study (described in full elsewhere^21^). This post-hoc analysis of ACE study data includes Nigerian subjects enrolled from January to February 2012 in general out-patient clinics of the National Hospital Abuja, University of Abuja Teaching Hospital Gwagwalada, Calabar General Hospital Cross River State Calabar, University of Nigeria Teaching Hospital Enugu, Federal Medical Centre Keffi Nassarawa State, General Hospital Akamkpa Cross River State and General Hospital Akpabuyo Cross River State. These sites were chosen specifically to reflect both urban and rural populations^21^. The Nigerian study sites were part of 94 out-patient general practice clinics in 14 countries (2337 out-patients from 8 African countries; 2041 out-patients from 6 Middle Eastern countries^21^).

Subject selection was from a primary care setting situated in urban and rural areas seeing patients of varied disease conditions and age groups. The rural populations are defined as isolated (distance of >50 km or lack of easy access to commuter transportation) from urban centers^22^. Although populations with a distance of < 50 km from urban centres may be rural, such rural study sites were not part of our study.

## Study procedure

Eligibility criteria were confirmed and demographic data including date of birth/age and gender were captured on a case report form (CRF). Medical history comprising all past/present diseases or syndromes that in the investigator's judgment were considered to be clinically significant with particular relevance to cardiovascular risk were recorded. Family history of premature coronary heart disease (CHD) was considered as positive if CHD occurred in a male first-degree relative aged <55 years and/or a female first-degree relative aged <65 years. CHD includes history of myocardial infarction, unstable/stable angina, coronary artery procedures (angioplasty or bypass surgery) or evidence of clinically significant myocardial ischemia.

All prior treatments administered for hypertension and dyslipidemia since diagnosis (if available/applicable) were recorded on the CRF. Other previous treatments considered clinically relevant were also recorded.

Patients were classified as a current smoker (consumed any cigarettes or tobacco, or used other forms of nicotine such as snuff, chewing tobacco, or any other local nicotine product during the past year), never smoked or ex-smoker. Age at smoking the first cigarette/nicotine product, number of cigarettes/nicotine products used per day and, if ex-smoker, when patient ceased smoking, were also recorded.

Patients were weighed, when wearing light clothing, using a calibrated scale and recorded to the nearest 0.1 kg. The scale's zero value was checked daily. Patient's height was measured with a height meter , where the patient removed his/her footwear and remained standing, with feet together on a horizontal, flat surface and heels, calves, glutei, spine and head in contact with the vertical edge of the height meter. The head was positioned such that the external auditory meatus and the external edge of the orbit formed a horizontal line. The movable part of the height meter was lowered toward the patient's head until it touched the top of the head. The patient then carefully moved away, and corresponding measurement was recorded to the nearest 0.5 cm.

Body mass index (BMI) (weight (kg)/[height in meters]^2^) was derived from the patient's measurements. A BMI ≥30kg/m^2^ was classified as obesity. Waist circumference was measured halfway between the inferior margin of the last rib and the crest of the ileum in the mid-axillary plane. Waist circumference was used to define abdominal obesity (men ≥ 94 cm, women ≥ 80 cm based on IDF consensus)^23^.

Arterial BP was determined after the patient had been sitting quietly for 5 minutes, using a standardized BP measuring instrument. Diastolic BP coincided with the phase V (disappearance of Korotkoff sound). BP was the average of two consecutive measurements, taken once on each arm. BP ≥140/90 mmHg was classified as hypertension. Heart rate was estimated by taking the radial artery pulse for 1 minute, after each arterial BP measurement. BP readings were classified according to the European Society of Cardiology (ESC) Guidelines^24^. Patients receiving BP-regulating drugs at the time of the study were classified as hypertensive regardless of the BP levels.

Fasting total cholesterol, high-density lipoprotein cholesterol (HDL-C), low-density lipoprotein cholesterol (LDL-C) and triglycerides were classified according to the US National Cholesterol Education Program Adult Treatment Panel (NCEP ATP) III Guidelines^25^. Fasting plasma glucose was classified per American Diabetes Association (ADA) guidelines^26^. Hence, six modifiable risk factors including dyslipidemia, hypertension, diabetes, smoking, BMI and abdominal obesity were determined. Subjects could withdraw from the study at any time, or they could be withdrawn at any time at the discretion of the investigator or sponsor for safety, behavioral or administrative reasons. If a subject did not return for a scheduled visit (i.e. blood draws at laboratory), every effort was made to contact the subject and document subject outcome. The investigator enquired about the reason for withdrawal, requested the subject to return for a final visit, if applicable, and followed-up with the subject regarding any unresolved adverse events. Subjects who withdrew and also withdrew consent had no further evaluations, and no additional data were collected. All study data were captured in a paper CRF.

## Inclusion and exclusion criteria

The full methodology for the ACE study has been reported elsewhere^21^. Male or female subjects aged >18 years who signed and dated the informed consent document were included while subjects who presented with any life-threatening disease/condition as well as pregnant women and/or lactating mothers were excluded.

## Sample size and sampling method

A total of 303 subjects were enrolled into the ACE study from Nigeria, and 301 completed. Participants were a subset of the multi-country ACE sample size which was determined by assuming that >90% of enrolled subjects contribute to the primary analyses, percentage of subjects with dyslipidemia and percentage of subjects with hypertension. The ACE study had a planned total sample size of 4300 subjects which permits the estimation of these percentages to within ± 1.6% with 95% confidence; i.e. the half-width of the 95% confidence interval (CI) will be 1.6 or less^21^. A minimum of 150 subjects were enrolled per country to obtain a sample size sufficient for exploring the primary endpoint within sub-groups (country, urban vs rural, age, etc.). Enrolment per country had a maximum number of subjects determined by the healthcare infrastructure and ability to provide subjects, based on pre-study feasibility assessment. In the full ACE study and this sub-analysis, every fifth patient seen on a particular day, fulfilling the inclusion and exclusion criteria, was included in the study.

## Data analysis

Analyses were primarily descriptive in nature. Binary data were summarized using the percent of subjects with the event and a 95% CI. Continuous data were reported using n, mean, standard deviation, median and range; a 95% CI for the mean was also computed. No interim analyses were planned. All statistical tests were two-sided and conducted at the 0.05 level of significance. P-values ≤0.05 were considered statistically significant. No adjustments for multiple testing were undertaken.

## Ethical approval

Approval of the ACE study protocol, protocol amendments and informed consent forms were obtained from the IRB/IEC as reported elsewhere^21^. The study was conducted in accordance with the Declaration of Helsinki on Ethical Principles for Medical Research Involving Human Subjects, adopted by the General Assembly of the World Medical Association (1996).

In addition, the study was conducted in accordance with the protocol, the International Conference on Harmonisation (ICH) guidelines on Good Clinical Practice (GCP), and applicable local regulatory requirements and laws. Protection of subject personal data was ensured and subject names were not included on any sponsor forms, reports, publications or in any other disclosures, except where required by laws. The informed consent form was in compliance with ICH GCP, local regulatory and legal requirements. The investigators ensured that each subject, or his/her legally acceptable representative, was fully informed about the nature and objectives of the study and possible risks^21^. The investigators obtained written, informed consent from each subject or the subject's legally acceptable representative before any study-specific activity was performed.

## Results

### Demographic characteristics

**Figure 1:**
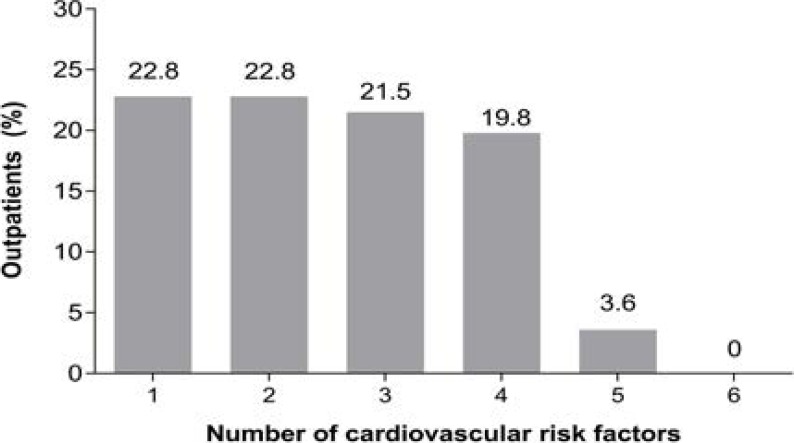
Proportion of out-patients presenting with one or more cardiovascular risk factors cardiovascular risk factors captured are: dyslipidemia, hypertension, obesity (defined as BMI ≥30 kg/m^2^), abdominal obesity, diabetes and smoking.

A total of 303 subjects (184 females [60.7%] and 119 males [39.3%]) from 8 out-patient general practice clinics in Nigeria were enrolled; 92 (30.4%) of the total cohort were enrolled from centers in rural communities. The mean age was 42.7 (± 13.1) years; about half (51.8%) were aged <45 years and only 12 (4%) were 65 years or older (Table 1). Overall, 138 subjects (45.5%) had 1–2 of the 6 modifiable cardiovascular risk factors measured. 65 (21.5%) had 3 risk factors; 19.8% had 4 risk factors, and 3.6% had 5 concurrent risk factors (Figure 1).

**Table 1:** Baseline parameters across the total Nigerian cohort and male and female populations

Variable	Total cohort (N=303)	Males (n=119)	Females (n=184)
Age	n (%)		
18–44 years	157 (51.8)	67 (56.3)	90 (48.9)
45–64 years	134 (44.2)	48 (40.3)	86 (46.7)
≥65 years	12 (4.0)	4 (3.4)	8 (4.3)
Mean years (range)	42.7 (18.0.85.0)	42.6 (19.0.85.0)	42.8 (18.0.82.0)
Rural	92 (30.4)	35 (38.0)	57 (62.0)
Urban	211 (69.6)	84 (39.8)	127 (60.2)
Median Systolic blood pressure (mm Hg)	132.0 (120.0, 145.0)	136.0 (120.0, 146.0)	130.0 (120.0, 144.0)
Median Diastolic blood pressure (mm Hg)	82.0 (74.0, 91.0)	86.0 (74.0, 93.0)	80.0 (74.0, 90.0)
Median Waist circumference (cm)	90.0 (82.8, 98.8)	88.5 (82.8, 96.5)	91.5 (82.2, 101.3)
Median BMI (kg/m^2^)	25.9 (23.0, 29.8)	24.7 (22.7, 28.1)	27.1 (23.4, 30.7)
Median Total cholesterol (mg/dL)	180.7 (154.4, 209.1)	175.5 (154.4, 204.6)	185.3 (162.2, 212.4)
Median LDL-C (mg/dL)	114.0 (86.1, 139.0)	108.1 (79.2, 135.1)	116.3 (94.1, 140.9)
Median HDL-C (mg/dL)	42.5 (33.9, 55.0)	42.5 (30.9, 56.1)	45.0 (34.7, 54.1)
Median Triglycerides (mg/dL)	97.4 (71.2, 132.8)	99.5 (69.0, 132.8)	97.4 (72.6, 132.8)
Median Fasting plasma glucose (mmol/L)	4.8 (4.2, 5.6)	4.8 (4.1, 5.4)	4.9 (4.2, 5.8)

Data are mean (%) or median (25^th^, 75^th^ percentile) unless otherwise indicated. BMI: body mass index; LDL-C: low-density lipoprotein cholesterol; HDL-C: high-density lipoprotein cholesterol.

### Dyslipidemia: prevalence

**Figure 2:**
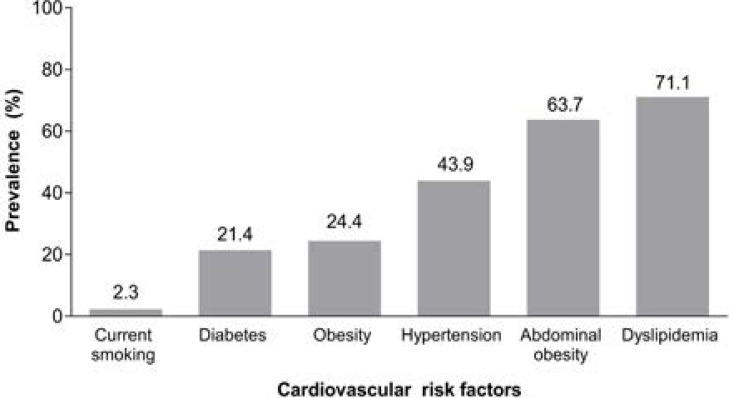
Overall prevalence of cardiovascular risk factors across Nigeria Graph to show the overall prevalence of dyslipidemia, hypertension, obesity (defined as BMI ≥30 kg/m^2^), abdominal obesity, diabetes and smoking across Nigeria

Dyslipidemia was the most prevalent cardiovascular risk factor recorded in 71.1% of 298 participants analysed (Figure 2). Median lipid values are given in Table 1. The most frequent type of dyslipidemia was low HDL-C, which was recorded in 117 participants (39%). Of the 303 enrolled subjects, 6 (2.0%) were diagnosed with dyslipidemia prior to the study, while screening identified an additional 206 (68.0%).

### Hypertension: prevalence

Elevated BP was recorded in 43.9% of 301 adult outpatients analyzed (Figure 2), where 114 had a prior history of hypertension and an additional 18 were found to have an abnormal BP reading at study encounter. Median systolic/diastolic BP was 132/82 mm Hg (Table 1). Of the 303 enrolled subjects, approximately one out of every 17 out-patients screened were found to have an elevated BP that was not known before (18 out-patients; 5.9%).

### Obesity: prevalence

Median (25th, 75th percentile) waist circumference was 88.5 cm (82.8–96.5) in men and 91.5 cm (82.2–101.3) in women, and median BMI was 25.9 kg/m^2^ (Table 1). The prevalence of obesity when defined by waist circumference (i.e., abdominal obesity), was appropriately 2.5 times more common than the prevalence of obesity when defined by BMI ≥30 kg/m^2^ (63.7% vs. 24.4% respectively) (Figure 2). Approximately 2 out of 3 out-patients screened had abdominal obesity (193 out-patients; 63.7%), and ∼1 out of every 4 out-patients screened had obesity (defined by BMI ≥30 kg/m^2^) (74 out-patients; 24.4%).

### Diabetes: prevalence

Approximately one-fifth of 280 adult out-patients analyzed had diabetes (Figure 2), with 40 of the 303 enrollees (13.2%) having a prior diagnosis and an additional 20 (6.6%) were diagnosed with an abnormal blood glucose level of ≥7 mmol/L. Out of the 303 enrolled subjects, approximately 1 out of 15 out-patients screened were newly diagnosed with diabetes (20 out-patients; 6.6%).

### Smoking: prevalence

The prevalence of current smoking was 2.3% of 303 subjects analyzed (6.3% past smokers) (Figure 2).

### Prevalence of risk factors by age, gender and community (urban vs. rural)

**Figure 3:**
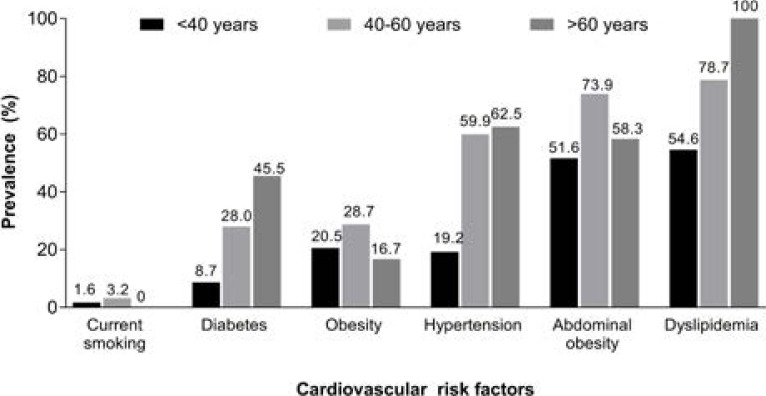
Prevalence of cardiovascular risk factors by age group

Older out-patients had higher rates of dyslipidemia, hypertension, and diabetes, while approximately 1/2 of the younger out-patients aged <40 years had dyslipidemia or abdominal obesity (Figure 3).

**Figure 4:**
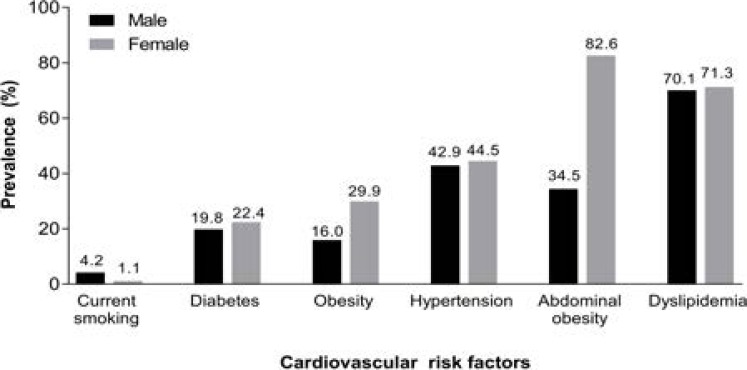
Prevalence of cardiovascular risk factors by gender

Male and females had similar prevalences of dyslipidemia, hypertension and diabetes but females had a higher prevalence of obesity and abdominal obesity. Smoking was more common in males (Figure 4).

**Figure 5:**
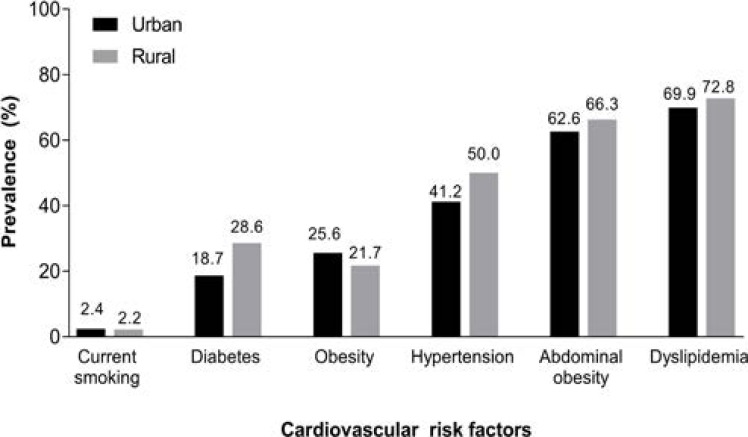
Prevalence of cardiovascular risk factors in rural and urban communities

The prevalence rates of most of the cardiovascular risk factors measured were higher in rural centers. Urban centers also demonstrate high rates of dyslipidemia (69.9%), hypertension (41.2%), diabetes (18.7%) and abdominal obesity (62.6%). Smoking prevalance was similar between rural and urban communities (Figure 5).

## Discussion

Primary prevention, early detection and prompt, effective control as well as health promotion strategies are critical in the attempt to reverse the global burden of CVD^27^,^28^. This cross-sectional study of Nigerian out-patients revealed a low prevalence of smoking (2.3%), but it importantly highlights the high prevalence of hypertension (43.9%) and the more alarmingly, the high prevalence of dyslipidemia (71.1%), most frequently associated with low HDL-C in almost 39% of patients. Furthermore, we report that obesity, defined either by BMI (24.4%) or in particular by abdominal circumference (63.7%) was also highly prevalent, possibly suggesting poor nutrition or an inadequate exercise culture among these Nigerian out-patients. Reorganization of the Nigerian food-pyramid, together with a national exercise incentive program may aid reducing the prevalence of obesity, targeting everywhere from homes to schools. Obesity and being overweight have been documented to start early in Nigerian pre-school and school children^30^,^31^, thus prompting the extension of control measures to age groups below those of the adult population in this study. Such a radical plan would need to be implemented by a multisectoral, multidisciplinary team involving communities, for example the Nigerian Labour Congress, women's organizations or religious groups^29^,^30^.

Dyslipidemia was also reported in more than two-thirds of adult out-patients in this cross-sectional study. The importance of reducing LDL-C levels by nutritional modification, exercise and careful deployment of statins cannot therefore be over-emphasized. Several studies in Nigeria have shown the presence of dyslipidemia in Nigerian patients with diabetes^31^–^33^ presenting with hypertension^34^ and in apparently normal health professionals^35^,^36^. The present study reveals the risk factors of obesity, hypertension, diabetes and dyslipidemia in not only young and old outpatients, both male and female, but also in out-patients in rural and urban areas where the rural–urban gap in obesity levels has narrowed and may, in some areas, have disappeared. Obesity remains more common in female Nigerian subjects attending adult general out-patient clinics however, perhaps pointing to inadequate post-natal exercise in Nigerian women.

The smoking prevalence was notably low in the present study of adult out-patients, aged 18 years and over. Although this is a positive finding it is somewhat in contrast to a global youth tobacco survey (GYTS) in 2008 in Nigeria which revealed a worryingly high prevalence of tobacco use among youths (boys and girls) — a serious phenomenon requiring aggressive intervention^37^. Smoking prevention and cessation guidelines in Africa and Middle East have been prepared and published^38^, and may have contributed to fewer adults continuing to smoke as found in our study.

Our study focused on six cardiovascular risk factors; multiple risk factors were found to be clustered in individuals, for example two risk factors in 22.8% of the subjects; three risk factors in 21.5%; four risk factors in 19.8%; and five risk factors in 3.6%. Our observations suggest better supervision and care of these risk factors are needed, particularly when they are found clustered in one individual. Without a concerted, multi-factorial approach with deliberate lifestyle modifications, an increase in tangible and intangible costs, deleterious effect on quality of life, and a heavier burden on national health infrastructures will occur. For example, the nationwide, Diabcare Nigeria Study (2008) demonstrated serious gaps in co-operation between patients and commitment from caregivers, with regard to meeting recommended targets for lipid, blood glucose, weight and BP, despite effective pharmacologic interventions and use of statins^39^. These collective observations highlight the importance of urgent, mandatory implication and adherence to international guidelines on cardiovascular disease management 25,26,40.

Comprehensive screening for risk factors in a large Nigerian population is, logistically and infrastructurally, a Herculean task; therefore, opportunistic screening, national and cultural education, mobilization of health-seeking attitudes and behavior throughout the population, alongside widespread implementation of WHO recommended surveillance^41^,^42^ of risk factors, are needed. National risk factor education is very important and should be maintained so that every teenager and adult knows his/her BP, weight, cholesterol, genotype and blood sugar level^43^, despite the socio-cultural complexities of such a venture^44^. Various screening studies in rural and urban Nigerian settings^45^–^47^ including house-to-house screening, as in Abia state NCD project^48^,^49^, have shown that these risk factors occur in both urban and rural areas; the old dichotomy is disappearing and may have in fact disappeared, as reported in South Africa^50^.

Even though income levels may create a barrier to screening programs^51^,^52^, because cost pressures will limit access, the use of primary healthcare centers is recommended. In a primary healthcare setting, high rates of physical inactivity (81.6%), diabetes (7.7%) and asymptomatic hypertension (26.4%) have been reported by others^53^, while among rural Fulani in Northern Nigeria, impaired fasting glucose (6.9%), impaired glucose tolerance (8%) and the effect of advancing age and obesity were observed^53^. In addition, the importance of screening young, pre-school and adolescents, in whom risk factors have been documented in Nigeria^55^, cannot be over-emphasized. It is important to note that this study was conducted in selected rural and urban hospital settings and the findings may not be generalized to the general population.

## Conclusion

Cardiovascular risk factors are highly prevalent in adults attending general out-patient clinics in Nigeria, many of whom were undiagnosed and therefore unaware of their cardiovascular risk status prior to screening. We recommend that opportunistic cardiovascular disease risk factor screening alongside intensive national, multisectoral cardiovascular education needs to be implemented, scaled up nationwide, and rolled out in both urban and rural communities in Nigeria.
